# Riparian and in-channel habitat properties linked to dragonfly emergence

**DOI:** 10.1038/s41598-020-74429-7

**Published:** 2020-10-19

**Authors:** Zoë G. O’Malley, Zacchaeus G. Compson, Jessica M. Orlofske, Donald J. Baird, R. Allen Curry, Wendy A. Monk

**Affiliations:** 1 Department of Biology, Canadian Rivers Institute, University of New Brunswick, 10 Bailey Dr., P.O. Box 4400, Fredericton, NB E3B 5A3 Canada; 2 Department of Biology, Environment and Climate Change Canada @ Canadian Rivers Institute, University of New Brunswick, P.O. Box 4400, Fredericton, NB E3B 5A3 Canada; 3 Department of Biological Sciences, University of Wisconsin-Parkside, 900 Wood Rd., P.O. Box 2000, Kenosha, WI 53141 USA; 4 Faculty of Forestry and Environmental Management, University of New Brunswick, 28 Dineen Dr., P.O. Box 4400, Fredericton, E3B 5A3 NB Canada; 5 Environment and Climate Change Canada @ Canadian Rivers Institute, Faculty of Forestry and Environmental Management, University of New Brunswick, P.O. Box 4400, Fredericton, NB E3B 5A3 Canada; 6 Present Address: Centre for Environmental Genomics Applications (CEGA), 14 International Pl. #102, St. John’s, A1A 0R6 NL Canada

**Keywords:** Freshwater ecology, Wetlands ecology, Riparian ecology

## Abstract

In freshwater ecosystems, habitat alteration contributes directly to biodiversity loss. Dragonflies are sentinel species that are key invertebrate predators in both aquatic (as larvae) and terrestrial ecosystems (as adults). Understanding the habitat factors affecting dragonfly emergence can inform management practices to conserve habitats supporting these species and the functions they perform. Transitioning from larvae to adults, dragonflies leave behind larval exoskeletons (exuviae), which reveal information about the emergent population without the need for sacrificing living organisms. Capitalizing on Atlantic Canada’s largest freshwater wetland, the Grand Lake Meadows (GLM) and the associated Saint John/Wolastoq River (SJWR), we studied the spatial (i.e., across the mainstem, tributary, and wetland sites) and temporal (across 3 years) variation in assemblages of emergent dragonflies (Anisoptera) and assessed the relative contribution of aquatic and terrestrial factors structuring these assemblages. The GLM complex, including the lotic SJWR and its tributaries and associated lentic wetlands, provided a range of riparian and aquatic habitat variability ideal for studying dragonfly emergence patterns across a relatively homogenous climatic region. Emergent dragonfly responses were associated with spatial, but not temporal, variation. Additionally, dragonfly communities were associated with both aquatic and terrestrial factors, while diversity was primarily associated with terrestrial factors. Specific terrestrial factors associated with the emergence of the dragonfly community included canopy cover and slope, while aquatic factors included water temperature, dissolved oxygen, and baseflow. Our results indicate that management of river habitats for dragonfly conservation should incorporate riparian habitat protection while maintaining aquatic habitat and habitat quality.

## Introduction

Dragonflies, which serve as important umbrella species in aquatic systems^1,2^, maintain dynamic functional roles as both predators and prey in terrestrial and aquatic environments^3,4^. Additionally, they can serve as important bioindicators for water quality^5–7^, mercury contamination^8^, ecological status^9,10^, and environmental change^11^. For these reasons, there have been many recent regional and international efforts aimed at conservation and recovery of these and other indicator insects^12,13^. Odonates require suitable habitat to facilitate emergence, and those species exhibiting synchronous emergence also require specific environmental cues^14,15^. Pressures, such as intensification of land use, flow management, and sea level rise, threaten dragonflies and their habitat^16–18^. Warming water temperatures may affect the timing of dragonfly larval emergence both indirectly, by altering thermal cues, and directly, by accelerating development, which in turn could have negative consequences for their ecological role in riparian habitats^19^. Therefore, a better understanding of the critical habitat and factors related to the larval stages of dragonflies is important for conserving and managing populations and their habitat.

Previous studies of dragonfly species have independently investigated the aquatic and terrestrial environmental conditions required for successful emergence. For example, the threatened Green Hawker (*Aeshna viridis*) requires a particular macrophyte species (the water soldier, *Stratioites aloides*) for protection from predators prior to emergence^20,21^. Further, low water pH can adversely affect the survival and emergence of other dragonflies^22^. Aquatic factors may also influence the transition to the adult stage, including optimal water temperature, which is necessary to synchronise mass emergence for some species^23^.

Terrestrial factors, such as riparian understory vegetation, trees, and canopy cover, have also been identified as important to the emergence of several species of dragonflies, including protected species^21,24,25^. For example, the Hungarian Balkan Goldenring (*Cordulegaster heros*), a threatened dragonfly, emerges primarily on riparian vegetation, specifically tree trunks in stream sections with 90% or more forest cover^14^. Riparian vegetation can also increase emergence success in other species by providing climbing structures and oviposition sites^25^. Therefore, both aquatic and terrestrial habitat characteristics are clearly important for larval development^25^, yet little attention has been given to exploring the combined influence of terrestrial and aquatic factors on whole communities of emerging dragonflies (but see Ref.^26^ for an examination of how aquatic and riparian factors influenced gomphids).

Investigations of dragonfly communities are aided by examination of exuviae, the final larval exoskeleton that often remains attached to riparian structures. Exuviae provide a physical record of their final larval condition, including information about their adult life form, enabling species identification and body size measurements^26–28^. Exuviae are an important tool for conservation studies because we can examine populations without having to collect or harm larval or adult individuals^24,29^ and avoid bias towards adult or larval stages that may not provide relevant knowledge of emergence success or critical emergence habitat^30^.

Here, we evaluate the relative influence of aquatic and terrestrial habitat factors on the spatial (between-habitat) and temporal (between-year) variation of the composition, diversity, abundance, and biomass of emergent dragonfly communities. Odonates spend most of their development in larval form in the aquatic environment but do use the structure of the riparian terrestrial environment during their transition to adult stage^31^. Given the extended period of larval development, we predicted that aquatic environmental factors would be more strongly associated with the composition, diversity, abundance and biomass of emergent dragonflies than terrestrial factors. Terrestrial factors were expected to have less influence on emergence relative to aquatic factors because dragonfly larvae are large, robust, armored, and capable of emerging on a variety of substrates^32^. We also predicted that dragonfly responses would be different between habitats (main channel, tributary, and wetland), but sites with similar environmental conditions would have similar responses. Finally, because our study occurred across a region with relatively consistent climate, we predicted dragonfly responses would not differ temporally. Addressing the interaction between environment and emergence responses, such as the factors affecting the timing and extent of secondary production, better informs management and conservation of dragonfly species, highlighting where to target restoration and conservation efforts.

## Methods

### Study area

**Figure 1:**
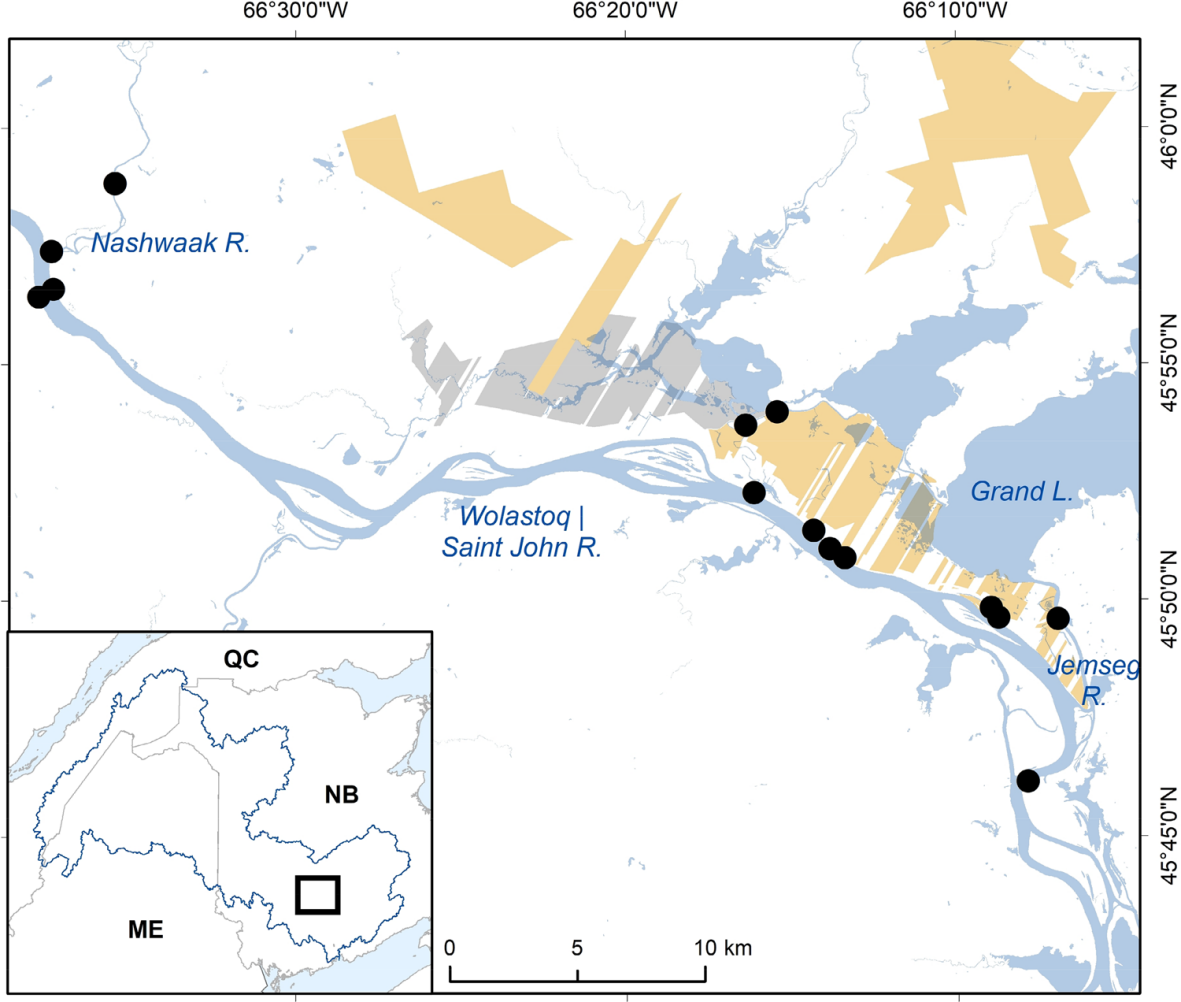
Map of all exuviae sample sites (*n* = 14) along a 44 km stretch of the lower Saint John/Wolastoq River watershed, including the Grand Lake Meadows and Nashwaak River, in New Brunswick, Canada. Most sites (*n* = 11) were surveyed from 2014 to 2016 and the remaining sites (*n* = 3) were surveyed in 2016.

This study took place in the lower Saint John/Wolastoq River (SJWR) watershed and associated Grand Lake Meadows (GLM) floodplain wetlands in central New Brunswick (Fig. 1). The SJWR is the longest inland river in the Maritimes at 673 km. The SJWR drainage covers 55,000 km^2^, and more than half of this area is within New Brunswick, with headwaters located in Quebec and Maine. Three hydropower dams on the mainstem (Beechwood, Mactaquac, and Grand Falls) regulate the river with additional structures on some of the major tributaries (e.g., Tobique). The GLM is located in the lower Saint John River floodplain, where it comprises the largest wetland complex in Atlantic Canada. This wetland contains protected habitat areas for conservation, including the federally protected Portobello Creek National Wildlife Area and the provincially protected GLM conservation area. The GLM contains some of the most productive habitat for wildlife in the province. The spring freshet each year floods the meadows and replenishes it with nutrients. The GLM also provides protection to surrounding residential areas from flooding, as well as habitat for many species including migratory birds, endangered plants, and dragonfly species. This freshwater system allowed us to explore benthic dragonfly communities in a variety of habitat types and flow regimes throughout the year. The mean annual temperature during the three years of study (2014–2016) varied between 5.50 and 6.32 °C (annual SD between 10.47 and 11.89 °C) and total annual precipitation varied between 829.5 and 1059.8 mm, based on data from a nearby long-term climate station.

### Field collection of exuviae

Exuviae were collected from fourteen sites in the GLM, including the federally protected Portobello Creek National Wildlife Area, along a 50 km stretch of the lower SJWR main channel in New Brunswick, Canada (Table 1; Fig. 1). Eleven sites in the lower SJWR and GLM were chosen to maximize the spatial variation within this area (hereafter called the GLM complex). Three sites (i.e., two located in the Nashwaak River and one in the GLM wetland area) were added in 2016 to include a broader range of site-level variables and habitat differences. The three different site classifications—river, wetland, and tributary—supported comparisons among habitat types (Table 1). Each site was divided into three subsites of equal length, designated as upstream, midstream, or downstream, in order to assess within site spatial variability.

**Table 1:** Dragonfly exuviae sample sites in the Grand Lake Meadows, Nashwaak River, and Saint John/Wolastoq River. Most sites (*n* = 11) were surveyed from 2014 to 2016, and the remaining sites (*n* = 3) were surveyed in 2016.

Site area	Site code	Habitat type	Years sampled
Upper Gagetown	26	Large river	2014–2016
Ararat Marsh	27	Large river	2014–2016
Upper Gagetown	32	Large river	2014–2016
McGowan’s Corner	33	Large river	2014–2016
McGowan’s Corner	34	Large river	2014–2016
Fredericton Northside	48	Large river	2014–2016
Fredericton Southside	51	Large river	2014–2016
Portobello NWA	29	Wetland	2014–2016
Thatch Island	31A	Wetland	2014–2016
Thatch Island	31B	Wetland	2014–2016
Jemseg	46	Wetland	2014–2016
Nashwaak	52	Tributary	2016
Nashwaak	53	Tributary	2016
The Oxbow	54	Wetland	2016

Flow types were classified based on the flow characteristics and water body of the site.

Sampling occurred from late May to early July in 2014, 2015, and 2016 to synchronously assess the majority of emerging riverine dragonfly species (excluding lentic and fall emerging species). Sites were visited multiple times throughout the sampling period (*n* = 4–5 times per site per year), with the goal of near census sampling all exuvia within the transects of each site. Sites were not sampled during storm events, which could have reduced exuviae detection. During each site visit, exuviae were collected by three observers (i.e., Z. O’Malley and two others) along a designated transect from the water's edge to the tree line. Transect interval widths varied with river water level, but was typically 10 m (range 0–20 m). Survey duration varied with numbers of exuviae present, and exuviae were exhaustively sampled. Transects ranged from 40 to 120 m in length, and included 13–41 trees. Each exuvia was placed in an individual container and the date of collection, site location, and substrate type (i.e., tree trunk, log, understory plant, grass, unattached) were noted. Exuviae were identified to species^31^, and body size was measured as the left fore-tibia, as the gross structure of exuviae deforms during drying after emergence^14,33,34^. A Leica M80 microscope (Leica, Wetzlar, Germany) at 10× magnification with an ocular micrometer was used to measure tibia lengths (mm).

### Habitat variables

The choice of measured habitat variables was based on hypothesized mechanistic linkages with dependent variables (e.g., the importance of water temperature as a cue for emergence in some species or the availability of potentially suitable emergence substrata; Table 2). All measured habitat variables were z-score standardized, and a predictor variable resemblance matrix with normalised environmental variables (*n* = 31) was used for analyses.

**Table 2:** Average measurements (standard deviation) for environmental variables recorded in 2016.

Variable	Large river	Wetland	Tributary
Slope (°)	8.86 (6.70)	12.80 (12.58)	9.83 (4.20)
Tree water distance (m)	4.54 (3.08)	1.67 (1.73)	6.01 (4.80)
Tree abundance	9.76 (4.44)	9.67 (3.89)	7.00 (4.00)
Tree diversity	0.65 (0.41)	0.52 (0.43)	0.54 (0.27)
Tree community	0.38 (0.87)	− 0.66 (0.71)	0.34 (0.87)
Canopy cover	0.92 (0.13)	0.94 (0.09)	0.91 (0.04)
Tree surface area (m^2^)	4.42 (1.41)	4.65 (2.44)	3.34 (0.95)
Tree density (per m^2^)	0.01 (0.01)	0.02 (0.02)	0.02 (0.02)
Bark depth (mm)	7.81 (2.26)	9.24 (1.89)	5.85 (1.28)
Bark roughness	0.25 (0.06)	0.24 (0.08)	0.38 (0.09)
Understory abundance	32.83 (15.47)	40.24 (12.58)	37.33 (15.01)
Understory diversity	0.59 (0.34)	0.96 (0.36)	0.95 (0.44)
Understory density (per m^2^)	22.76 (10.73)	27.91 (8.72)	25.89 (10.41)
Understory cover (%)	50.59 (35.90)	50.89 (18.83)	33.77 (35.24)
Dissolved oxygen (%)	92.18 (3.31)	81.51 (10.12)	89.97 (4.44)
Conductivity (µS/cm^3^)	97.71 (19.41)	73.33 (35.56)	60.00 (0.00)
Salinity (units)	0.05 (0.01)	0.03 (0.01)	0.03 (0.00)
pH	6.52 (0.42)	6.57 (0.50)	7.02 (0.12)
Velocity (m/s)	0.02 (0.04)	0.04 (0.09)	0.08 (0.09)
3-Day maximum (cm)	96.68 (46.79)	102.33 (14.59)	85.43
Baseflow (cm)	0.59 (0.14)	0.47 (0.17)	0.85
Number of reversals	7.83 (0.98)	4.67 (1.15)	7.00
Macrophyte cover (%)	12.62 (15.70)	17.67 (28.46)	0.00 (0.00)
Substrate size (mm)	1.71 (1.97)	0.60 (0.96)	4.78 (4.70)
Embeddedness (%)	74.60 (31.89)	76.11 (27.97)	73.61 (28.10)
Median diurnal range (°C)	2.97 (1.92)	2.26 (0.81)	2.01
Degree days	553.50 (24.17)	600.07 (56.84)	558.99
Temperature (°C)	18.25 (1.71)	19.23 (1.48)	20.32 (0.10)

Loggers were deployed at ten sites (large river = 6, wetland = 3 and tributary = 1). Note that standard deviation could not be reported for the tributary water depth and water temperature variables because of the single logger within the category.

Terrestrial habitat variables included the tree community, the understory community, and associated metrics of these communities, including % cover, density, tree position (i.e., distance to water), tree diameter at breast height (DBH, ~ 1.3 m), tree surface area (from root crown to 4 m up the tree, or the tree survey area), and tree bark roughness. Tree canopy cover (%) was measured using a spherical crown densiometer at each sub-site, halfway from the water to the closest tree. The tree community, which was dominated by maple (i.e., *Acer rubrum*, *Acer saccharinum*, *Acer saccharum*) was assessed by identifying all trees at each site and recording the abundance of each species. The understory plant community and associated variables were assessed using images (*n* = 3 per sub-site) captured by a Nikon Coolpix camera held 1 m above the ground (1.04 × 1.39 m) and positioned halfway from the water to the closest tree at the same points as the tree canopy cover measurements. Images were then assessed to determine percent cover by assessing the proportion of points intersecting vegetation from a regular grid (*n* = 96 points) superimposed on each image; vegetation from each image was also morphotyped and counted. While bark roughness has been described categorically in other studies^35^, we quantitatively examined the variation in bark roughness, defined as the standard deviation of bark depth measurements (*n* = 9) made from the surface of a plastic 20 × 20 cm template wrapped around each tree at breast height (i.e., higher deviations indicated higher bark roughness values). Additionally, slope was measured at each site using a clinometer at three points corresponding to three random trees. Tree and understory vegetation communities were described using non-metric multidimensional scaling (NMDS) ordination scores.

Aquatic habitat variables included macrophyte cover (mean of two independent estimates), substrate size (measured using the Wolman 100-pebble count method^36^), and substrate embeddedness (%, recorded after every tenth measurement). The following water quality point measurements were recorded at each sub-site using a YSI 556 Multiprobe System multiparameter water quality meter (YSI, Yellow Springs, OH, USA) and Marsh McBirney Flo-Mate 2000 electromagnetic velocity flow meter (Hach, Loveland, CO, USA): velocity (m/s), depth (cm), dissolved oxygen (%), salinity (units), conductivity (µS/cm^3^), and pH. Finally, we deployed programmable HOBO Onset U20L loggers (Onset, Cape Cod, MA, USA) for water pressure and HOBO pendant loggers (Onset, Cape Cod, MA, USA) for water temperature to help quantify the site-level variability in both water depth (m) and temperature (°C) over a longer period. Logger data were truncated to June 3–July 26, 2016 to account for slight timing differences in deployment and retrieval among sites. Data were checked for errors, including potential stranding with water level changes, and corrected using adjacent sites where needed. Thirty-three water level-based variables were calculated using the Indicators of Hydrologic Alteration (IHA) software (V 7.1)^37^, and three variables (baseflow (cm), three-day max depth (cm), and the number of reversals) were selected based on cross-correlation analysis. Two water temperature variables were calculated including the total degree days during the logger deployment and the median diurnal range of temperatures.

### Abundance, biomass, and diversity metrics

Dragonfly abundance was the sum of individual exuviae of each species collected during each sampling event over the specified sampling period. Dragonfly biomass was calculated using biomass estimates from regression models: equations were developed by regressing the left fore tibia length of exuvia against the total length within each family (*n* = 5)^14^. Total length was then used to convert to larval dry biomass using length-mass relationships for each family obtained in previous studies^38,39^. Dragonfly diversity was represented using Shannon’s index (*H*) calculated using PC-Ord^40^. Dragonfly, tree, and understory assemblages (see details on how vegetation assemblages were assessed below) were compared using nonmetric multidimensional scaling (NMDS) ordinations using Bray–Curtis distances in PC-Ord^40^. A Principal Coordinates Analysis (PCoA) was used via the *vegan* package^41^ in R^42^ to visualize both spatial and temporal assemblage variation by grouping samples by site nested in habitat and year nested in habitat.

### Spatiotemporal aspects of emergence

Spatiotemporal variability in dragonfly emergence (2014–2016) was analysed using permutational multivariate analysis of variance (PERMANOVA) models within the *vegan* package^41^ in R^42^. Separate models were created for each dragonfly response variable: abundance, biomass, species assemblage, and diversity. Each of these variables were normalised and Euclidian distance measures were used for resemblance matrices. Community matrices were log_10_ (*x* + 1) transformed and the Bray Curtis distance measure was used for the resemblance matrices. All PERMANOVAs used type III sums of squares and were run for 999 permutations with a critical significance level of 0.05^43,44^.

Spatial variation in dragonfly responses was determined at the site level, where dragonfly exuvial assemblage metrics were determined within each sub-site in 2016. Spatial analyses included habitat characteristics and all three habitat types (i.e., main channel, wetland, and tributary). The PERMANOVA models were two factor designs with Site (14 levels, fixed) and habitat type (three levels, fixed). The same resemblance matrices for abundance, biomass, and diversity response variables were used as for the temporal analyses. The dragonfly assemblage consisted of 25 species (Supplementary Table 1).

Temporal analyses were conducted at the tree level where dragonfly exuviae community metrics were assessed at each tree, in order to ensure sufficient statistical power to support hypothesis testing. Two habitat types (main channel and wetland) were included but habitat-level characteristics were not assessed in the temporal analyses. Temporal PERMANOVA models included four factors: Year (three levels, fixed), Flow Type (two levels, fixed), Site (11 levels, random) and Tree (269 levels, random). Replication was at the tree level across seven large river sites and four wetland sites. The dragonfly assemblage consisted of 33 riverine species (Supplementary Table 1).

### Modeling the factors influencing dragonfly emergence

Partial Mantel tests (10,000 permutations per test) were performed using the *ecodist* package^45^ in R^42^ to control for spatial distance among sites while assessing associations among environmental variables and dragonfly response variables. Geographic distance was calculated using a spatial distance matrix for all 2016 sites from which Euclidean distances were calculated. Environmental predictors consisted of z-score standardized aquatic and terrestrial factors that were grouped together respectively to simplify model operation. Separate models were created for each of the four dragonfly responses (abundance, biomass, diversity and assemblage). Because increasing the number of variables in partial Mantel models is known to inflate type I error rates^46^, we chose to only assess high-level models using this approach and applied false discovery rate corrections to path *p*-values within each model to account for multiple comparisons^47^. In order to assess the associations between specific environmental variables and dragonfly responses, we developed distance-based linear models (DistLM) in PRIMER 7^48^ using a step-wise procedure and Akaike Information Criterion with corrections (AICc) to find the best fitted model even with a large number of predictor variables. For partial Mantel and DistLM models, we examined the dragonfly communities using both raw and log_10_ (*x* + 1) transformed data.

## Results

### Summary of exuvial collections

**Figure 2:**
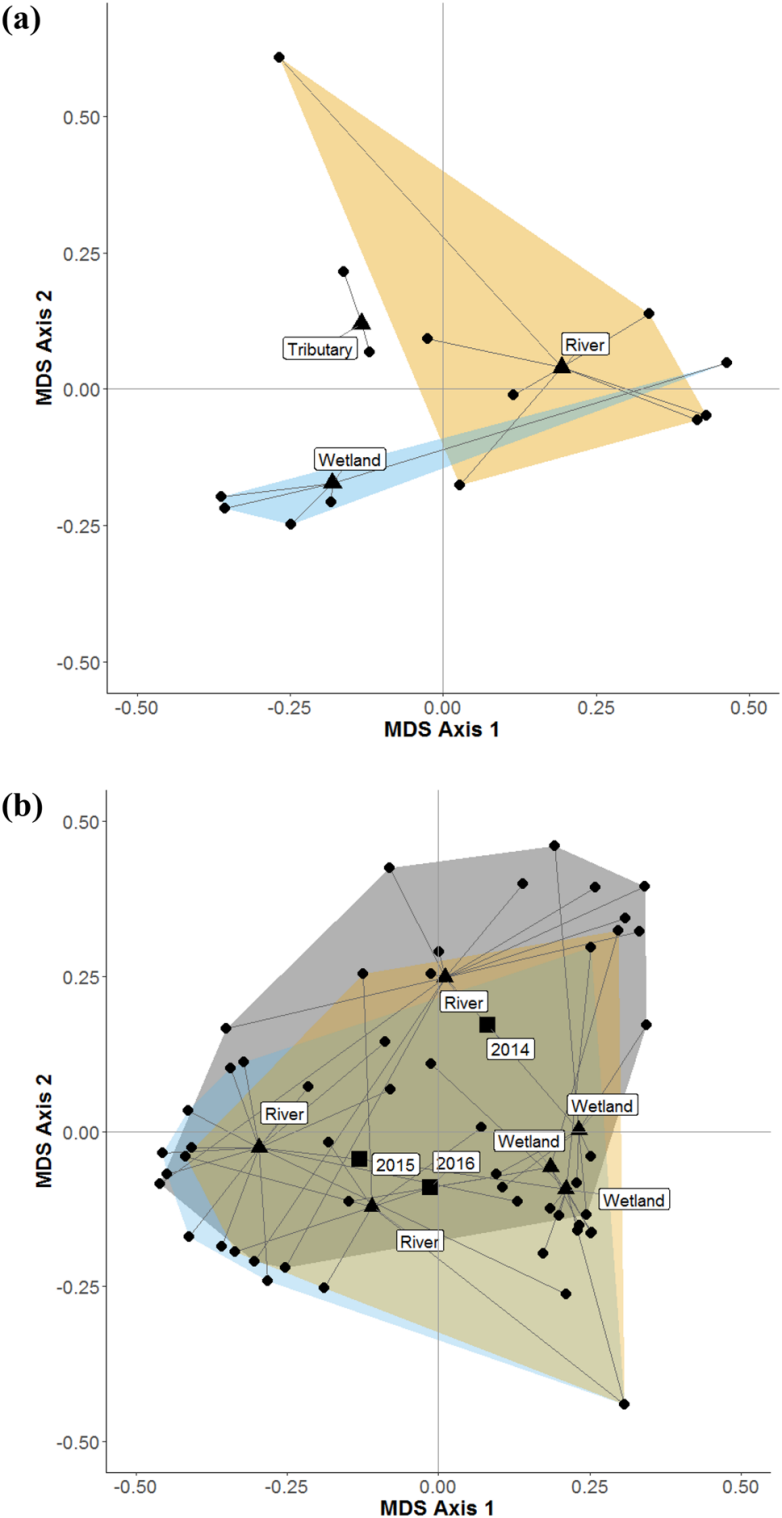
Principal Coordinates Analysis (PCoA) of distances among centroids of Bray–Curtis dissimilarity PCoA scores for emergent dragonfly communities at (**a**) 14 sites within three habitat types sampled in 2016 only and (**b**) 11 sites within two habitat types sampled between 2014 and 2016. Circles represent individual sites, triangles represent habitat type, and squares represent year. Polygons are coloured by (**a**) habitat type and (**b**) year.

Separate datasets were created to complete the spatial (2016) and temporal (2014–2016) analyses (Table 1, Fig. 2). For the spatial analyses, 66 exuviae were collected in 2016 on average per site across the 14 sites, with a range of 1–1360 individuals and an average of five species found at each site (range 1–23). *G. vastus* comprised 81% of the samples, while *G. ventricosus* and *T. spinigera* comprised 1.6% and 4.7%, respectively (Table 1). For the temporal dataset, dragonfly exuviae were collected across 11 sites for the 2014–2016 emergence seasons (Table 1). On average, 100 exuviae (range 2–787) across six species (range 1–12) were collected per site per year. The four most abundant species made up ~ 84% of the total sample from 2014 to 2015. *Gomphus vastus* represented 72% of all individuals, while *G. ventricosus*, a local species-at-risk, comprised 5%. *Didymops transversa* and *Tetragoneuria spinigera* each accounted for 2.8% of specimens.

### Spatiotemporal factors affecting dragonfly emergence

Dragonfly responses (abundance, biomass, diversity, and assemblage structure) from 2014 to 2016 were examined to determine temporal effects at the tree level (*α* = 0.05). None of the response variables were significantly different across years or between large river and wetland sites (Fig. 2b). The response variables were all significantly different across sites and demonstrated a significant year and site interaction (all *p* < 0.05 except for diversity).

Spatial analysis at the site level revealed that emergent dragonfly abundance, biomass, and diversity were not significantly different among the main channel, wetland, and tributary habitat types in 2016. However, dragonfly assemblages differed significantly at the habitat level (*p* < 0.01) (Fig. 2a). Pairwise tests showed that there were significant differences between main channel and wetland assemblages (*t* = 1.77*, p* = 0.034) and between main channel and tributary assemblages (*t* = 1.73*, p* = 0.024). Assemblage structure did not differ significantly between wetland and tributary habitats (*t* = 1.41*, p* = 0.095).

### Relative influence of aquatic and terrestrial factors affecting dragonfly emergence

**Figure 3:**
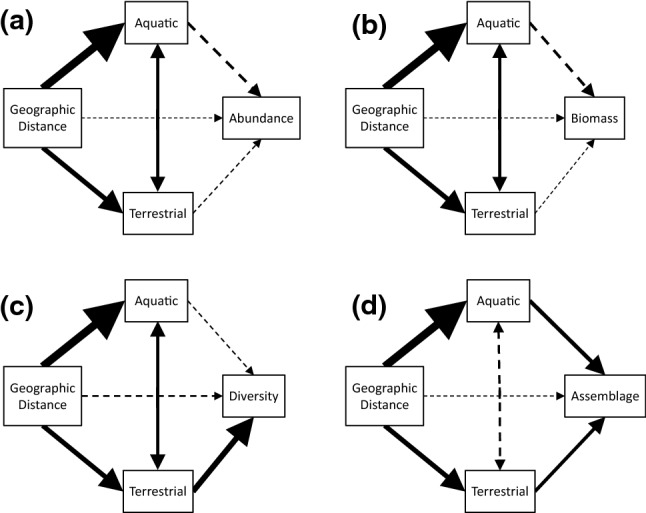
Partial Mantel tests for environmental effects in 2016 on exuvial dragonfly (**a**) abundance, (**b**) biomass, (**c**) diversity, and (**d**) assemblage. The thickness of the arrows indicates the relative Mantel *r* values, where thicker arrows are larger values and thinner arrows are smaller values. Solid arrows had a *p* value < 0.05, and dashed arrows had a *p* value > 0.05 after False Discovery Rate adjustments for multiple comparisons.

Partial Mantel models revealed no significant effects of aquatic or terrestrial variables on emerging larval abundance (aquatic: Mantel *r* = − 0.13, *p* = 0.92; terrestrial: Mantel *r* = 0.06, *p* = 0.41; Fig. 3a) or total biomass (aquatic: Mantel *r* = − 0.13, *p* = 0.92; terrestrial: Mantel *r* = 0.04, *p* = 0.46; Fig. 3b). Terrestrial habitat variables, however, did affect dragonfly diversity (Mantel *r* = 0.27, *p* = 0.0014), but aquatic habitat variables did not (Mantel *r* = − 0.06, *p* = 0.91; Fig. 3c). Moreover, there was a significant effect of both aquatic (Mantel *r* = 0.19, *p* = 0.00035) and terrestrial (Mantel *r* = 0.16, *p* = 0.0011) habitat variables on the dragonfly assemblage (Fig. 3d); log_10_ (*x* + 1) transforming dragonfly community data did not change these patterns (aquatic: Mantel *r* = 0.19, *p* = 0.0012; terrestrial: Mantel *r* = 0.16, *p* = 0.0063).

**Figure 4:**
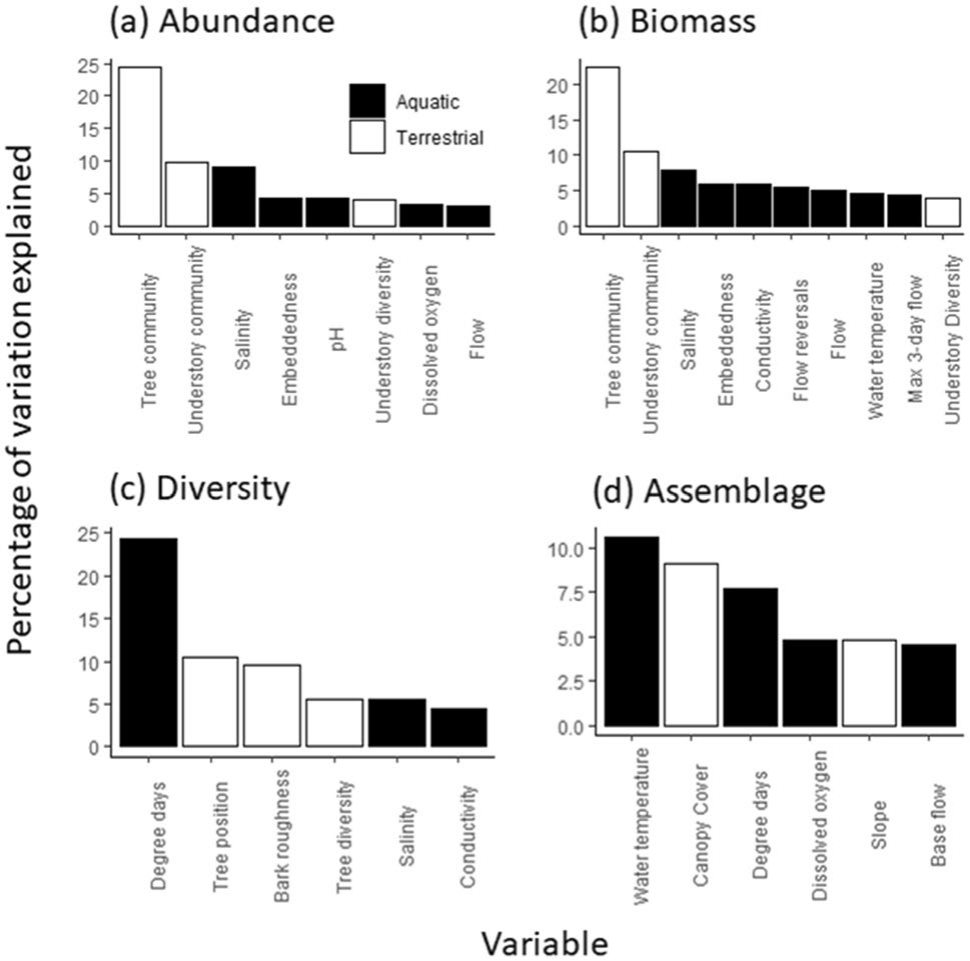
Distance-based linear models on dragonfly responses in 2016 including (**a**) abundance, (**b**) biomass, (**c**) diversity, and (**d**) assemblage. Variables for each response were based on the best solution for each model, with the percentage of variation explained for each variable.

Dragonfly abundance and biomass DistLM models explained 71% and 76% of the variation in dragonfly responses, respectively. Variation in abundance and biomass was driven primarily by two terrestrial factors: tree community (24% and 22%, respectively) and understory community (10% for both variables) (Fig. 4a,b; Supplementary Table 2). Salinity explained 9% of the variation in abundance and 8% of the variation in biomass. The dragonfly diversity DistLM model explained 60% of the variation, and the response was driven by aquatic degree days (24%), with tree position and bark roughness both explaining 10% of the variation (Fig. 4c; Supplementary Table 2). Overall, aquatic variables explained 34% and terrestrial variables explained 26% of the variation in dragonfly diversity. Only 41% of the variation in the dragonfly assemblage was explained by the DistLM model: water temperature (11%), canopy cover (9%), and aquatic degree days (8%) explained the most variation (Fig. 4d; Supplementary Table 2). Overall, more variability was explained by aquatic variables (28%) than terrestrial variables (14%) in the assemblage DistLM model. Log_10_ (*x* + 1) transforming the dragonfly assemblage data did not substantially influence the DistLM model, as the top four most important variables (water temperature, canopy cover, aquatic degree days, and dissolved oxygen) were the same (total model variation explained: 39%; Supplementary Table 2).

## Discussion

### Spatiotemporal patterns of dragonfly emergence

Patterns of dragonfly emergence did not change between 2014 and 2016. These findings contrast with a previous study in Hungary that found abundance and species composition in emergent riverine dragonfly populations, especially gomphids, changed from year to year^23^. The discrepancy in temporal patterns in that study compared to our study was likely due to a difference in scale: our study examined a relatively small spatial scale (~ 340 km^2^ area encompassing all sites, 44.1 km maximum distance between farthest sites; average = 19.03 km, SD = 14.45 km), whereas the other study examined 700 km of river^23^. However, this other study also found that dammed sites had less temporal variation in abundance and species composition^23^, corroborating the results of our study, where nine of our mainstem sites were downstream of a hydropower generating station, possibly homogenising temporal differences among sites. Collectively, these findings have implications for river management: regulated river flows may affect the variation in emergence while more dynamic, unregulated rivers may create more variation in dragonfly emergence patterns. Additionally, larval cohort splitting could lead to greater temporal variation in abundance when portions of a population emerge at different times^27,49,50^. Cohort splitting can occur for species that have multi-year development, as variation in larval development could necessitate another year of growth prior to emergence for some individuals^23,50^. Furthermore, split cohorts may emerge in different areas due to separation from the major cohort, influencing abundance and species composition at measured emergence sites^49^.

Dragonfly assemblage was the only emergence response that was significantly different among habitat types in 2016, with river sites differing significantly from wetland and tributary sites. A primary difference between the wetland and tributary sites compared to the main channel sites was the amount of flow regulation, which has been shown to cause spatial differences in assemblages of emerging insects in other systems^23,51^. While our dragonfly communities were dominated by gomphids (Supplementary Table 1), log_10_ (*x* + 1) transforming our data down-weighted abundant taxa, rendering the observed patterns more reflective of the entire community rather than these dominant taxa.

Despite supporting unique dragonfly assemblages, the abundance, biomass, and diversity of emergent dragonflies were not different among our sites, indicating that the productivity and diversity of all habitat types was similar. Larvae could be using different microhabitats or habitat types depending on their feeding strategy, affecting the types of dragonflies present but not the abundance and biomass of dragonflies. For example, Gomphidae are riverine specialists that require appropriate substrates suitable for burrowing. In previous studies, one of the species collected in our surveys, *Ophiogomphus mainesis*, showed a preference for cobble and coarse substrates^52^. Aeshnidae, which are typically lentic, are reported to be more dependent on aquatic vegetation^53^; however, some riverine aeshnids also show an affinity for particular substrate sizes^52^. Various dragonflies may also use terrestrial environments differently during and after emergence. Consistent with previous reports, the exuviae of *Didymops transversa*, a member of the Macromiidae, was often observed furthest from the water’s edge (> 8 m), suggesting that in some cases individuals of this species traverse a variety of terrestrial habitats before selecting an emergence site^31^. While Gomphidae are reported to emerge from horizontal substrates^31,54^, most of our records of *Gomphus vastus and Gomphus ventricosus* were collected on vertical substrates (e.g., trees). It was, however, typical to retrieve other members of the family, notably *Arigomphus furcifer*, from horizontal substrates, often very close to the edge of the water. Following emergence, many Gomphidae begin to forage in tree canopies, while sexually mature Libellulidae remain near the water and perch on emergent aquatic and riparian vegetation to patrol feeding areas and protect territories^53^. These differing strategies could influence the density and area emergent dragonflies occupy, as well as the predators targeting them. For instance, riparian spiders may be able to catch dragonflies that patrol in the riparian area, while birds would be able to access the canopy-dwelling dragonflies and those in flight^55–58^. Varied dragonfly habitat uses and associated strategies of their predators could also influence the resulting nutrient subsidies^9^ and functions^1^ dragonflies provide to the riparian ecosystem.

### Relative influence of aquatic and terrestrial factors affecting dragonfly emergence

Emergence is a period of high risk for dragonflies. During this period, they are susceptible to predation^59,60^. Limited emergence substrate due to overcrowding of co-emerging specimens can also lead to insufficient space for successful emergence, yielding mortality rates of 16%^61^. Physical factors can influence dragonfly emergence either directly, by impacting the process of emergence, or indirectly, by providing emergence shelter or structure. For example, wind and cold temperatures are important physical factors that can impede emergence^53,59^ or disrupt synchronization cues^62^. We predicted that terrestrial habitat factors would have less influence on dragonfly emergence relative to aquatic factors. We found that terrestrial factors contributed to dragonfly diversity, and aquatic and terrestrial factors both contributed to dragonfly community composition, indicating that both factors are important to dragonfly emergence, albeit in different ways. Because log_10_ (*x* + 1) transforming our dragonfly assemblage matrices affected neither partial Mantel tests nor significant predictors indicated by DistLM models, these patterns likely represent community-wide responses rather than responses from a few gomphid taxa.

Our results demonstrate that terrestrial factors had a strong influence on shaping the diversity and assemblages of emerging dragonflies within habitats of the SJWR and GLM complex in our study. Tree position, bark roughness, and tree diversity were significantly associated with the diversity of dragonflies, while canopy cover and slope were significantly associated with the assemblage of emerging dragonflies. Terrestrial habitat factors may require considerable time to establish, and, once removed, may be difficult to restore^63^. Trees require years or decades of growth to mature, and riparian vegetation is associated with increased habitat complexity^64^ and quality^65,66^, as well as the abundance and diversity of associated communities^67,68^. Trees provide habitat structures to support the successful emergence of dragonflies, and are likely favored over other emergence structures because they are stable and tall enough to reduce interference from fluctuating water levels^53,69^. Tree trunks also provide a large surface area for adult emergence, which likely reduces intraspecific competition for emergence sites^69^ by allowing for adequate spacing during emergence, which could reduce damage during molting^61^ and reduce density-dependent predation^70,71^. Trees with greater bark roughness, which increases the surface area of trees and provides varied microhabitats for emergence, tend to be favored over trees with smooth bark^72^. Additionally, riparian canopy cover can provide shade and habitat structure in the immediate emergence area, while the slope of a site is likely linked to other factors, such as bank steepness and depositional dynamics, which were not considered here. Riparian vegetation and canopy cover may attract the larvae of certain dragonfly species to an emergence location, providing structure and shelter for dragonfly emergence, thereby influencing dragonfly abundance and shaping community assemblages^24,25,73^. Consequently, preserving the immediate riparian habitat, which protects dragonflies from predation and other physical factors, is particularly important where protected species occur.

Aquatic factors that influenced emergent dragonfly assemblages in our study included water temperature, degree days, dissolved oxygen, and baseflow. In contrast to terrestrial factors influencing emergence, aquatic habitat variables can be more variable, but overall patterns could affect dragonfly larvae prior to emergence. Water temperature and dissolved oxygen provide emergence cues and suitable aquatic habitat prior to emergence^62,74^. Alterations to cues like water temperature can affect the onset and timing of emergence^62,75,76^, which can lead to differing species assemblages^15^. Canopy cover, a terrestrial factor associated with the dragonfly community, also affects local water temperature via shading^77^. Future changes to water temperature and salinity beyond certain thresholds could create environments that dragonflies can no longer tolerate, leading to poor growth conditions and asynchronous or early emergence, and ultimately lower reproductive success and survival^75,76^. Furthermore, anthropogenic effects, such as global climate change and land use changes affecting the thermal regime, can influence the onset of dragonfly emergence and species distributions^75,76,78^, potentially disrupting reciprocal subsidy linkages from aquatic insect emergence^19,55,56,79,80^. Long-term aquatic habitat conditions (e.g., baseflow) are particularly important for dragonflies in temperate climates, since each annual cohort must overwinter to emerge the next season, with some species overwintering for multiple years before emergence^50,54^. Understanding the environmental factors affecting emergence will help inform management and conservation of dragonfly species during every stage of their life history.

Freshwater habitats and their riparian zones are increasingly threatened by a combination of climate change-driven extreme events and ongoing human development^81,82^, impacts that could affect the dragonfly community and subsequently affect higher trophic levels in both aquatic (e.g., fish) and terrestrial (e.g., spiders, amphibians, insectivorous birds) ecosystems. Therefore, understanding the relative contribution of terrestrial and aquatic habitat factors on aquatic insect emergence is necessary for proper management and conservation. Conserving species and communities is facilitated by a holistic understanding of the relative influence of habitat conditions throughout development, particularly for biphasic organisms like dragonflies. Our results emphasize the importance of focusing conservation efforts on both riparian and aquatic habitats simultaneously, a charge that was born out of an early appreciation of vital aquatic-terrestrial linkages^83,84^, but one that is seldom heeded in practice, especially at the landscape scale^85^.

## Supplementary information

Supplementary Tables.

## Data Availability

All data, R code, metadata, and supplemental information associated with this manuscript are available via the GitHub project repository, which can be found here: https://github.com/zacchaeus-compson/Habitat-factors-linked-to-dragonfly-emergence.
