# Supplementary material for: Riparian and in-channel habitat properties linked to dragonfly emergence
Source: Sci Rep. 2020 Oct 19;10:17665. doi: 10.1038/s41598-020-74429-7 (PMC7573607; doi:10.1038/s41598-020-74429-7)
Supplement: Supplementary file 1 — Supplementary Tables. [file 41598_2020_74429_MOESM1_ESM.docx]

# Riparian and in-channel habitat properties linked to dragonfly emergence

Zoë G. O’Malley^1^, Zacchaeus G. Compson^1,2,^*, Jessica M. Orlofske^3^, Donald J. Baird^2^, R. Allen Curry^1,4^, and Wendy A. Monk^5^

^1^ Canadian Rivers Institute, Department of Biology, University of New Brunswick, 10 Bailey Dr., P.O. Box 4400, Fredericton, NB, E3B 5A3, Canada

^2^ Environment and Climate Change Canada @ Canadian Rivers Institute, Department of Biology, University of New Brunswick, P.O. Box 4400, Fredericton, NB, E3B 5A3, Canada

^3^ Department of Biological Sciences, University of Wisconsin-Parkside, 900 Wood Rd., P.O. Box 2000, Kenosha, WI, 53141, U.S.A.

^4^ Faculty of Forestry and Environmental Management, University of New Brunswick, 28 Dineen Dr., P.O. Box 4400, Fredericton, NB, E3B 5A3, Canada

^5^ Environment and Climate Change Canada @ Canadian Rivers Institute, Faculty of Forestry and Environmental Management, University of New Brunswick, P.O. Box 4400, Fredericton, NB, E3B 5A3, Canada

*corresponding author

## Supplementary Information

Supplementary Table 1: List of all dragonfly species found on trees in 2014 – 2016 in the Grand Lake Meadows complex for this study. Asterisks depict species that were found across the site in 2016 including trees and understory.

| **Species name** | **Family** | **2014** | **2015** | **2016** |
| --- | --- | --- | --- | --- |
| *Basiaeschna janata*(Say, 1840) | Aeshnidae | 1 | 1 | 1* |
| *Cordulegaster maculata* (Sélys, 1854) | Cordulegastridae | 1 | 0 | 0 |
| *Didymops transversa*(Say, 1839) | Macromiidae | 1 | 1 | 1* |
| *Epitheca princeps*(Hagen, 1861) | Corduliidae | 0 | 0 | 1* |
| *Epitheca spinosa* (Hagen in Sélys, 1878) | Corduliidae | 0 | 0 | 0* |
| *Macromia illinoiensis*(Walsh, 1862) | Corduliidae | 1 | 1 | 1* |
| *Neurocordulia michaeli*(Brunelle, 2000) | Corduliidae | 1 | 1 | 0* |
| *Neurocordulia obsoleta*(Say, 1839) | Corduliidae | 1 | 1 | 0 |
| *Neurocordulia yamaskanensis*(Provancher, 1875) | Corduliidae | 1 | 1 | 1* |
| *Somatochlora williamsoni*(Walker, 1907) | Corduliidae | 0 | 0 | 0 |
| *Epitheca canis*(McLachlan, 1886) | Corduliidae | 0 | 1 | 1* |
| *Epitheca cynosura*(Say, 1839) | Corduliidae | 1 | 1 | 1* |
| *Epitheca semiaquea*(Burmeister, 1839) | Corduliidae | 0 | 1 | 1* |
| *Epitheca spinigera* (Sélys, 1871) | Corduliidae | 0 | 1 | 1* |
| *Arigomphus furcifer* (Hagen in Sélys, 1878) | Gomphidae | 1 | 1 | 0 |
| *Dromogomphus spinosus*(Sélys, 1854) | Gomphidae | 0 | 0 | 1* |
| *Gomphus abbreviatus* (Hagen in Sélys, 1878) | Gomphidae | 1 | 1 | 1* |
| *Gomphus adelphus* (Hagen in Sélys, 1878) | Gomphidae | 1 | 1 | 1* |
| *Gomphus aspersus*(Morse, 1895) | Gomphidae | 0 | 1 | 0 |
| *Gomphus borealis* (Needham, 1901) | Gomphidae | 1 | 1 | 0* |
| *Gomphus descriptus*(Banks, 1896) | Gomphidae | 1 | 0 | 0 |
| *Gomphus exilis*(Sélys, 1854) | Gomphidae | 1 | 1 | 0* |
| *Gomphus spicatus* (Hagen in Sélys, 1854) | Gomphidae | 1 | 1 | 1* |
| *Gomphus vastus* (Walsh, 1862) | Gomphidae | 1 | 1 | 1* |
| *Gomphus ventricosus* (Walsh, 1863) | Gomphidae | 1 | 1 | 1* |
| *Hagenius brevistylus*(Sélys, 1854) | Gomphidae | 0 | 0 | 0* |
| *Ophiogomphus carolus* (Needham, 1897) | Gomphidae | 0 | 1 | 0* |
| *Ophiogomphus colubrinus*(Sélys, 1854) | Gomphidae | 1 | 1 | 1* |
| *Ophiogomphus mainensis*(Packard in Walsh, 1863) | Gomphidae | 0 | 1 | 0* |
| *Ophiogomphus rupinsulensis*(Walsh, 1862) | Gomphidae | 1 | 1 | 1* |
| *Ophiogomphus aspersus*(Harvey, 1898) | Gomphidae | 0 | 1 | 0 |
| *Stylogomphus albistylus*(Hagen in Sélys, 1878) | Gomphidae | 0 | 0 | 0* |
| *Leucorrhinia intacta* (Hagen, 1861) | Libellulidae | 1 | 0 | 0 |

Supplementary Table 2: DistLM of environmental variables on dragonfly responses in 2016. The variables were based on the best solution for the given model.

| **Response** | **AICc** | **Total variability explained (%)** | **Predictors** | **Variability explained (%)** | ***Pseudo-F*** | ***p*** | **+/-** |
| --- | --- | --- | --- | --- | --- | --- | --- |
| Abundance | -29.54 | 71.12 | Tree community | 24.38 | 12.89 | 0.001 | + |
|  |  |  | Understory community | 9.76 | 5.78 | 0.019 | + |
|  |  |  | Salinity | 9.02 | 6.03 | 0.022 | + |
|  |  |  | Embeddedness | 4.32 | 3.29 | 0.087 | + |
|  |  |  | Flow | 3.11 | 2.81 | 0.079 | + |
|  |  |  | Understory diversity | 4.10 | 4.04 | 0.053 | + |
|  |  |  | Dissolved oxygen | 3.25 | 3.26 | 0.081 | + |
|  |  |  | pH | 4.18 | 4.65 | 0.038 | + |
| Biomass | -29.89 | 75.85 | Tree community | 22.35 | 11.51 | 0.002 | + |
|  |  |  | Understory community | 10.45 | 6.07 | 0.018 | + |
|  |  |  | Salinity | 7.89 | 5.05 | 0.033 | + |
|  |  |  | Conductivity | 5.93 | 4.11 | 0.051 | + |
|  |  |  | Water temperature | 4.51 | 3.32 | 0.080 | + |
|  |  |  | Embeddedness | 5.94 | 4.84 | 0.032 | + |
|  |  |  | Understory diversity | 3.96 | 3.46 | 0.089 | + |
|  |  |  | Flow | 5.05 | 4.91 | 0.034 | + |
|  |  |  | Max 3-day flow | 4.36 | 4.72 | 0.043 | + |
|  |  |  | Flow reversals | 5.42 | 6.96 | 0.014 | + |
| Diversity | -22.20 | 60.00 | Degree days | 24.39 | 12.91 | 0.003 | + |
|  |  |  | Tree position | 10.49 | 6.28 | 0.014 | + |
|  |  |  | Bark roughness | 9.52 | 6.50 | 0.016 | + |
|  |  |  | Tree diversity | 5.56 | 4.11 | 0.046 | + |
|  |  |  | Conductivity | 4.49 | 3.55 | 0.062 | + |
|  |  |  | Salinity | 5.54 | 4.85 | 0.037 | + |
| Community | 343.49 | 41.35 | Water temperature | 10.56 | 4.72 | 0.001 | + |
|  |  |  | Canopy cover | 9.07 | 4.40 | 0.001 | + |
|  |  |  | Degree days | 7.68 | 4.01 | 0.001 | + |
|  |  |  | Dissolved oxygen | 4.79 | 2.61 | 0.005 | + |
|  |  |  | Slope | 4.76 | 2.71 | 0.004 | + |
|  |  |  | Base flow | 4.50 | 2.69 | 0.004 | + |
| Community* | 337.25 | 39.15 | Water temperature | 11.68 | 5.29 | 0.001 | + |
|  |  |  | Canopy Cover | 10.06 | 5.01 | 0.001 | + |
|  |  |  | Dissolved oxygen | 7.80 | 4.21 | 0.001 | + |
|  |  |  | Degree days | 5.26 | 2.99 | 0.003 | + |
|  |  |  | Understory community | 4.35 | 2.58 | 0.004 | + |

*Designates log_10_ (*x*+1) transformed variable.
